# Changes in mental health among Chinese university students before and during campus lockdowns due to the COVID-19 pandemic: a three-wave longitudinal study

**DOI:** 10.3389/fpsyt.2023.1267333

**Published:** 2023-11-14

**Authors:** Ying Qing, Zhiyan Li, Yuhang Zhang

**Affiliations:** ^1^Student Innovation Center, Shanghai Jiao Tong University, Shanghai, China; ^2^Key Laboratory for the Genetics of Developmental and Neuropsychiatric Disorders, Ministry of Education, Bio-X Institutes, Shanghai Jiao Tong University, Shanghai, China; ^3^School of Environmental Science and Engineering, Shanghai Jiao Tong University, Shanghai, China; ^4^School of Life Sciences and Biotechnology, Shanghai Jiao Tong University, Shanghai, China

**Keywords:** COVID-19, campus lockdown, mental health, university students, longitudinal study

## Abstract

The campus lockdown due to the COVID-19 pandemic has adversely affected mental health among university students. However, the heterogeneity in responses to campus lockdown is still poorly known. We collected three-wave prospective data on university students’ mental health in Shanghai, China, in 2022: (i) in February before the pandemic; (ii) in April at the initial COVID-19 campus lockdown; and (iii) in May amidst the citywide lockdown. Overall, 205 university students completed sociodemographic questionnaires, the General Health Questionnaire-12 items (GHQ-12), and the Depression, Anxiety and Stress Scale-21 items (DASS-21). Generalized estimating equations were used to examine the longitudinal changes in mental health and symptoms of depression, anxiety, and stress. Latent class mixed models (LCMM) were constructed to identify distinct trajectories. Multinomial regression models were used to identify factors associated with status variation patterns. Mean GHQ-12 scores were 8.49, 9.66, and 11.26 at pre-pandemic and lockdown T1 and T2, respectively (*p* < 0.001). Mean scores for depression, anxiety, and stress were (5.96, 10.36, and 8.06, *p* < 0.001), (7.13, 6.67, and 7.16, *p* = 0.243), and (9.83, 7.28, and 11.43, *p* < 0.001), respectively. Changing trends of numbers of participants with clinical symptoms were consistent with those of mean scores. LCMM fitted three distinct trajectory classes, respectively, for GHQ-12, depression and anxiety symptoms, and four classes for stress symptoms. Participants with fair or poor peer relationships were more likely to belong to vulnerable trajectories concerning depression, anxiety, and stress symptoms. This study proves heterogeneity in mental health of university students in response to pandemic campus lockdown and highlights the necessity for identifying vulnerable groups to provide targeted support in future pandemics.

## Introduction

Since the World Health Organization (WHO) declared the outbreak of the SARS-CoV-2 coronavirus (COVID-19) a pandemic on 11 March 2020, the virus and consequent lockdown measures have negatively affected public mental health. Among the general population, university students were at a higher risk for mental distress during the COVID-19 pandemic (1–6) and appeared particularly susceptible to the adverse impacts of campus lockdown (7, 8), leading to significant changes in students’ lives, such as social isolation, uncertainty about the professional and academic career, and financial constraints (9–12).

The evolution of the COVID-19 pandemic is undetermined and may have long-term effects on mental health. Recent evidence has shown that psychological responses to the COVID-19 pandemic are heterogeneous (13, 14). Nevertheless, whether mental health responses to the pandemic lockdown among university students are heterogeneous remains to be clarified, which has limited our understanding of the specific effect of the campus lockdown due to the pandemic on mental health. Although extensive cross-sectional studies worldwide have shown deteriorating mental health, elevated symptoms of depression, anxiety, and stress among university students during the COVID-19 crisis (1, 15–17), there is still a lack of longitudinal data comparing the same populations before and during the pandemic lockdowns, making it difficult to interpret the exact changes in mental health among university students in response to the COVID-19 lockdowns. A few longitudinal studies have demonstrated worsened symptoms of depression, anxiety, and stress among university students before and during the COVID-19 lockdown (18), whereas others have reported relatively unchanged mental health (19), or only deterioration in depression but not anxiety (20) or increased symptoms for anxiety but not for depression (21). These findings have implied that responses to the COVID-19 lockdown are heterogeneous, not homogeneous, among university students. Accordingly, identifying subgroups of students with different trajectories of change in mental health to provide them with efficient help and intervention is needed.

Notably, studies on the distinct trajectories of mental health conditions before and across different lockdown phases among university students are still lacking. Further, exploring the sociodemographic factors involved in the distinct trajectories of mental health status is also warranted to identify university students more vulnerable to the adverse effects of the COVID-19 crisis and supply key targets for interventions.

Hence, we conducted a study of the evolution of mental health and emotional well-being among university students over the two lockdown phases in Shanghai, using a pre-pandemic cohort to overcome the scarcity of longitudinal studies including pre-pandemic data. This study aimed to investigate longitudinal changes in mental health among university students before and during the COVID-19 pandemic, identify the potential distinct trajectories of general mental health and symptoms for depression, anxiety, and stress, and explore the associations between sociodemographic factors and mental health trajectory classes.

## Methods

### Study design and data collection

Data were collected as part of a longitudinal survey study investigating university students’ mental health, belonging to the Participation in Research Program of Shanghai Jiao Tong University (SJTU) (No. T080PRP41017). The authors assert that all procedures contributing to this work comply with the ethical standards of the relevant national and institutional committees on human experimentation and with the Helsinki Declaration of 1975, as revised in 2008. Participants were recruited from SJTU students through online advertisements using convenience sampling method. A three-wave online survey was used to assess the effect of the campus lockdown due to the COVID-19 pandemic on the general mental health and emotional well-being of university students.

The first wave of data was collected on 26–27 February 2022 (baseline pre-pandemic data) with 228 participants completing surveys; the second wave on 8–13 April 2022 (lockdown T1) with 227 participants completing surveys, when campus lockdown measures had been introduced for approximately 4 weeks at SJTU and Shanghai had implemented citywide quarantine measures for around 1 week; the third wave on 21–27 May 2022 (lockdown T2) with 213 participants completing surveys, when SJTU had been locked down for over 10 weeks and Shanghai began to ease the quarantine measures. A total of 205 participants completed three-wave data collection.

### Measures

#### Sociodemographic information

Sociodemographic characteristics including age, gender, height, weight, major, grade, family residence, number of children in the family, parents’ education level, annual household income, and smoking and drinking habits, were collected. Additionally, self-reported peer relationships were also recorded with a score of 0–10.

#### Mental health outcomes

The General Health Questionnaire-12 items (GHQ-12) (22), validated within a Chinese population (23), was employed to assess the recent mental health status of university students. Each item is graded on a 0 to 4 Likert scale: 0, not at all; 1, no more than usual; 2, rather more than usual; or 3, much more than usual. A total score derived for each wave is calculated as the sum of each item, ranging from 0 to 36, and a score of >15 is considered to experience psychosocial malaise (24). In addition to the total score used to generate a mean score, a bimodal GHQ scoring method (0–0–1–1) is derived to identify those reporting clinical mental distress with a threshold ≥4. Cronbach’s alpha of the measure is 0.862.

The Depression, Anxiety, and Stress Scale-21 items (DASS-21) (25), validated within Chinese university student populations (26, 27), measured the emotional well-being of university students. This scale comprises 21 items grouped into three subscales: depression, anxiety, and stress. Each item is rated on a 4-point Likert scale: 0, never; 1, sometimes; 2, often; 3, almost always. Each subscale contains 7 items, and a total subscale score, ranging from 0 to 42, is calculated by summing the scores of the relevant items and then multiplying the sum by 2. Scores of >9, > 7, and > 14 are considered to represent depression, anxiety, and stress symptoms, respectively. The corresponding Cronbach’s alpha of the measure is 0.805, 0.712, and 0.790, respectively.

#### Sample size

The sample size was determined based on a previous prospective longitudinal study that investigated the impact of the COVID-19 outbreak on the mental health of undergraduate students, where the authors employed the DASS-21 to assess variations in depression, anxiety, and stress scores (21). To detect an effect size of 0.8 at a significance level of 5% with 80% statistical power, assuming a standard deviation of 7.86, a minimum of 121 participants was required.

### Statistical analysis

The Shapiro–Wilk test was performed to examine the normality of continuous variables. A Chi−Square test was conducted to compare categorical variables in mental health-related characteristics among three waves. Generalized estimating equations (GEEs) were performed to analyze three-wave longitudinal data of GHQ-12 and DASS-21 to determine the effect of campus lockdown due to COVID-19 pandemic on mental health. GEEs were selected due to their capacity for population-averaged inference, ability to model non-Gaussian distributions, and consideration of within-subject correlation. Latent class mixed models (LCMM) were constructed to identify distinct trajectories of GHQ-12 and DASS-21 depression, anxiety, and stress symptoms before and during the pandemic lockdowns using the R “lcmm” package (28). These models included fixed effects for time and discrete random variables for the latent classes and were fitted with one to 5 latent classes. Each model with two or more classes used random starting values from the model with the 1-class and a grid search function with 50 iterations was used to avoid the model identifying local maxima. Model fit was determined using the Bayesian information criterion (BIC), the sample size-adjusted Bayesian information criterion (SABIC), a measure of entropy, higher posterior probabilities of class membership, and interpretability. Besides, the results for LCMM followed the Guidelines for Reporting on Latent Trajectory Studies (29). Multinomial regression models were fitted to investigate associations between sociodemographic characteristics and distinct trajectories of each health outcome, and the adjusted odds ratio (aOR) and 95% confidence interval (CI) were used to represent the potential correlations. A *p* < 0.05 was considered statistically significant. All analyses were performed using R 4.1.0.

## Results

### Descriptive statistics

In total, this study included 205 university students (mean age = 19.4 years) who had data available for all three phases (pre-lockdown, lockdown T1, and lockdown T2). Among these participants, 97 were female, 179 were undergraduates, and 107 were majoring in engineering. Detailed sociodemographic characteristics are summarized in Table 1.

**Table 1:** Demographic characteristics of the study population.

Variables	N (%)
Students	205 (100)
Gender	
Male	108 (52.68)
Female	97 (47.32)
BMI	
Underweight ≤18.5	25 (12.20)
Normal weight 18.5–24.9	157 (76.59)
Overweight 25–29.9	21 (10.24)
Obesity ≥30	2 (0.97)
Smoking habits	
Yes	1 (0.49)
No	204 (99.51)
Drinking habits	
Yes	184 (89.76)
No	21 (10.24)
Grade	
Undergraduate	179 (87.32)
Postgraduate	26 (12.68)
Major	
Engineering	107 (52.20)
Liberal arts	14 (6.83)
Science	63 (30.73)
Medicine	21 (10.24)
Family residence	
North	80 (39.02)
South	125 (60.98)
Only-child family	
Yes	134 (65.37)
No	71 (34.63)
Peer relationships	
Poor 0–3	4 (1.95)
Fair 4–7	89 (43.42)
Good 8–10	112 (54.63)
Father’s education level	
Junior high school or below	43 (20.98)
High school	44 (21.46)
College or above	118 (57.66)
Mother’s education level	
Junior high school or below	45 (21.95)
High School	58 (28.29)
College or above	102 (49.76)
Annual household income (CNY)	
<80,000	44 (21.46)
80,000–300,000	124 (60.49)
>300,000	37 (18.05)

**Figure 1:**
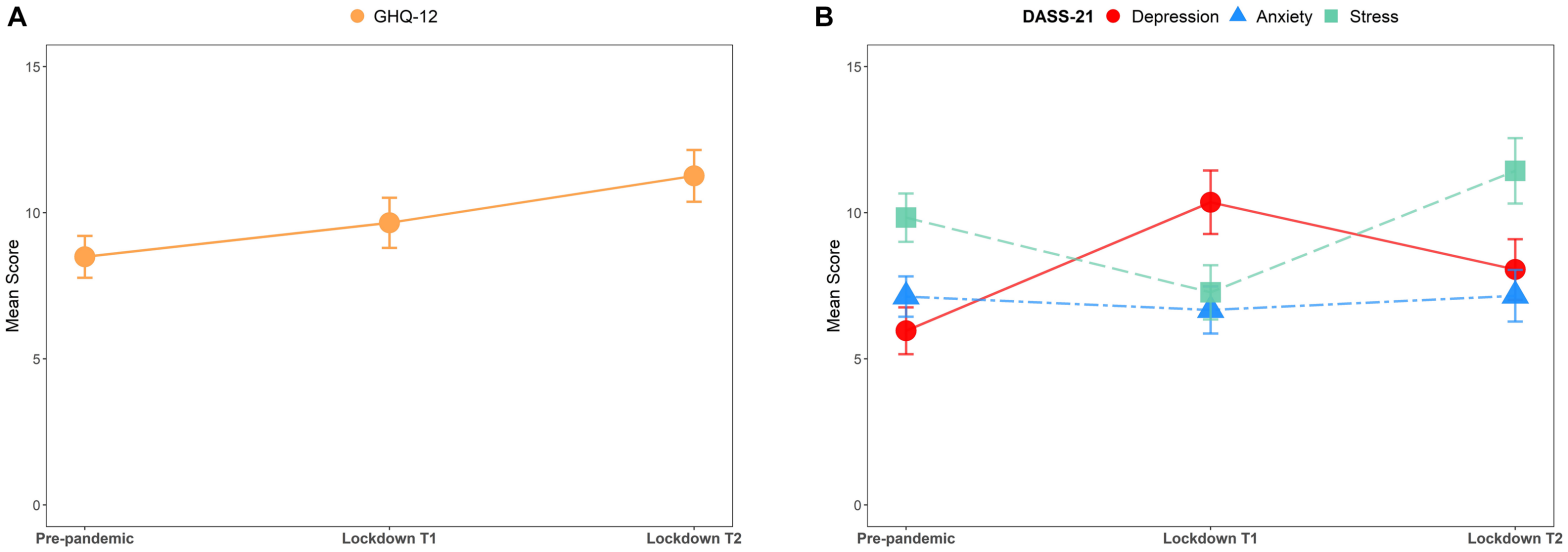
Mean GHQ-12 and DASS-21 scores over three data collection waves before and during the COVID-19 pandemic. **(A)** GHQ-12 scores (mean ± 95% CI) in each period. **(B)** DASS-21 scores (mean ± 95% CI) for depression (red), anxiety (blue), and stress (green) in each period.

Figure 1 and Table 2 show the descriptions of mental health outcomes before the pandemic and during lockdowns T1 and T2. Mean GHQ-12 scores progressively increased in a stepwise manner from pre-pandemic (8.49) to lockdown T1 (9.66) to T2 (11.26) (Figure 1A), suggesting that the mental health status of university students worsened in response to campus lockdowns amid the COVID-19 pandemic. Furthermore, the mean DASS-21 depression scores improved sharply from 5.96 during the pre-pandemic period to 10.36 at lockdown T1 but declined to 8.06 at lockdown T2 (Figure 1B). In contrast, the mean DASS-21 stress scores decreased from 9.83 during the pre-pandemic period to 7.28 at lockdown T1, and then increased to 11.43 at lockdown T2 (Figure 1B). The mean DASS-21 anxiety scores were relatively stable across the three periods (Figure 1B). Additionally, the changing trends in the number of participants experiencing depression, stress, or anxiety symptoms were in line with the trends observed in the mean scores for the three subscales (Table 2). These results showed that depression and stress but not anxiety symptoms of university students were significantly affected by the abrupt onset of COVID-19 pandemic and campus lockdown durations, and that depression symptoms changed in stark contrast to stress symptoms before and during the pandemic lockdowns.

**Table 2:** Descriptions of mental health outcomes across three phases.

Variables	Pre-pandemic	Lockdown T1	Lockdown T2	*p*-value
	Mean (SD)	Mean (SD)	Mean (SD)	
GHQ-12	8.49 (5.20)	9.66 (6.23)	11.26 (6.44)	< 0.001^a^
DASS-21 depression	5.96 (5.82)	10.36 (7.89)	8.06 (7.52)	< 0.001^a^
DASS-21 anxiety	7.13 (5.01)	6.67 (5.86)	7.16 (5.99)	0.243^a^
DASS-21 stress	9.83 (5.99)	7.28 (6.75)	11.43 (8.13)	< 0.001^a^
	N (%)	N (%)	N (%)	
Mental distress				<0.001^b^
No	169 (82.44)	144 (70.24)	124 (60.49)	
Yes (GHQ-12 score ≥ 4)	36 (17.56)	61 (29.76)	81 (39.51)	
Depression symptoms				<0.001^b^
No	163 (79.51)	92 (44.88)	130 (63.41)	
Yes (DASS-21 depression score > 9)	42 (20.49)	113 (55.12)	75 (36.59)	
Anxiety symptoms				0.832^b^
No	127 (61.95)	129 (62.93)	114 (55.61)	
Yes (DASS-21 anxiety score > 7)	78 (38.05)	76 (37.07)	91 (44.39)	
Stress symptoms				<0.001^b^
No	168 (81.95)	178 (86.83)	143 (69.76)	
Yes (DASS-21 stress score > 14)	37 (18.05)	27 (13.17)	62 (30.24)	

^a^Generalized estimating equation models. ^b^Chi-square test.

### Trajectories of mental health outcomes

**Figure 2:**
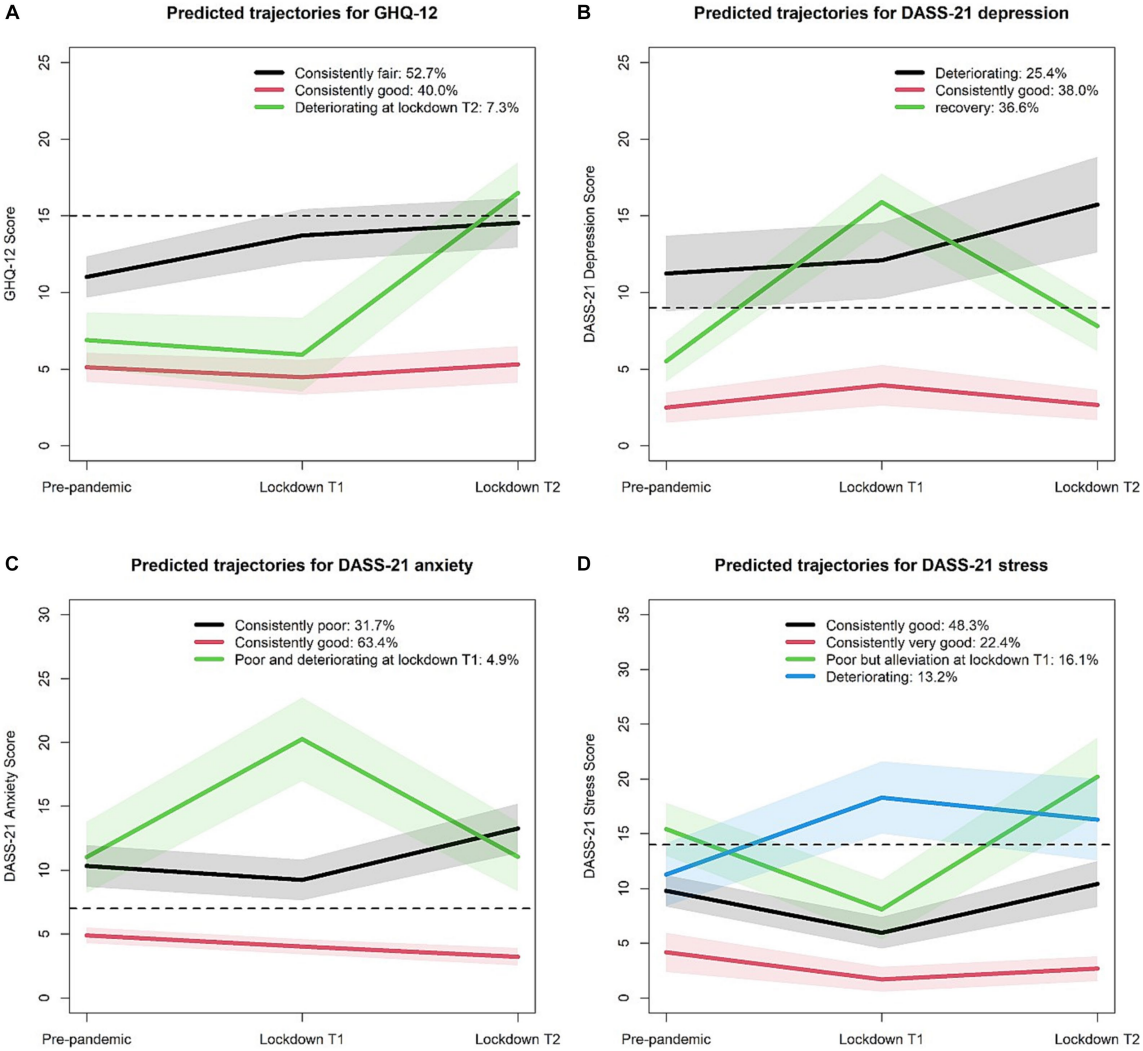
Mean scores for GHQ-12 and DASS-21 from class-specific trajectories across three waves of data collection before and during the pandemic. Mean scores for GHQ-12 **(A)**, DASS-21 depression **(B)**, and DASS-21 anxiety **(C)** from 3-class trajectories and for DASS-21 stress **(D)** from 4-class trajectories across three periods. Shaded ribbons represent the 95% CI for each period, and dashed lines indicate the clinical symptom cutoff scores.

LCMM models were fitted with up to five latent classes, and the goodness of fit indices is presented in Supplementary Table S1. The 3-class model was selected as the optimal solution for GHQ-12 scores, DASS-21 depression scores, and DASS-21 anxiety scores, while the 4-class model was chosen for DASS-21 stress scores. Figure 2 shows the identified class-specific trajectories of the observed scores for GHQ-12 and DASS-21.

Of the total participants, 40% belonged to the “consistently good” class, while 52.7% were in the “consistently fair” class, representing good or fair mental health status over time (Figure 2A). Additionally, 7.3% of the participants followed a “good but deteriorating at lockdown T2” trajectory, with a good pre-pandemic status, then a slight improvement from pre-pandemic to lockdown T1, but a sharp deterioration from lockdown T1 to T2.

Regarding depression symptoms, 3 distinct DASS-21 depression trajectories emerged (Figure 2B). A proportion of 38.0% of the participants belonged to the “consistently good” class, showing no clinical depression symptoms. A “deteriorating” group (25.4% of the participants) had initial clinical depression symptoms before the pandemic, and reported a sustained deterioration in depression symptoms over lockdown durations. Another “recovery” group showed no clinical depression symptoms prior to the pandemic, worsened depression symptoms at the initial lockdown, and then returned to around pre-pandemic levels of DASS21 depression scores at lockdown T2.

Most of the participants (63.4%) had no clinical anxiety symptoms across pre-pandemic and lockdown periods (Figure 2C), belonging to the “consistently good” class. A “consistently poor” group (31.7% of the participants) had pre-pandemic clinical anxiety symptoms that were sustained with high scores after campus lockdown. A “poor and deteriorating at lockdown T1” group (4.9% of the participants) showed worsened anxiety symptoms across the three periods and reported a sharp deterioration in anxiety symptoms at the initial shock of the pandemic lockdown.

Regarding stress symptoms, most of the study population belonged to either “consistently good” (48.3% of the participants) or “consistently very good” (22.4% of the participants) class across pre-pandemic to lockdown T2, with little divergence from their pre-pandemic scores (Figure 2D). A proportion of 16.1% of the participants belonged to “poor but alleviation at lockdown T1,” with an initial worsened status, then an alleviation at lockdown T1, and then a sharp deterioration at lockdown T2. A “deteriorating” group (13.2% of the participants) had a good status before the pandemic but a sharp deterioration at lockdown T1 and remained high scores at T2.

### Factors associated with mental health trajectories

To explore factors associated with distinct mental health trajectory classes, multinomial regression models were fitted. The associations between sociodemographic characteristics and distinct trajectories of GHQ-12 scores are shown in Supplementary Table S2. Participants with fair or poor peer relationships or those with father’s education levels of junior high school or below were more likely to belong to the “consistently fair” class rather than the “consistently good” class. Participants with an annual household income exceeding 300,000 CNY were more likely to develop psychosocial malaise at lockdown T2 and less likely to belong to the “consistently fair” class compared to the “consistently good” class.

For the trajectory classes of DASS-21 depression, participants with fair or poor peer relationships were more likely to belong to the “deteriorating” or “recovery” class than the “consistently good” class (Supplementary Table S3).

Supplementary Table S4 displays the associations between sociodemographic characteristics and latent classes for DASS-21 anxiety scores. Participants with fair or poor peer relationships were more likely to develop anxiety symptoms.

Concerning the trajectory classes of DASS-21 stress (Supplementary Table S5), participants with fair or poor peer relationships were more likely to belong to the “deteriorating,” “poor but alleviating at lockdown T1,” or “consistently good” classes than the “consistently very good” class. Participants whose father’s education level was at or below junior high school were more likely to belong to the “deteriorating” class than the “consistently very good” class.

## Discussion

After the worldwide COVID-19 pandemic over 3 years ago, the Shanghai government announced the citywide lockdown on April 1, 2022 to stem the outbreak and spread of the pandemic. Before the citywide quarantine measures were implemented, universities in Shanghai had locked down the campuses and confined students to their dormitories to prevent the spread of COVID-19 in March 2022. This study took advantage of a three-wave longitudinal survey to identify disparate trajectories of mental health among university students across before and during the COVID-19 campus lockdowns and to explore factors associated with these mental health trajectories. The findings contribute to our understanding of how pandemic campus lockdowns specifically impact the mental health of university students and underscore the importance of identifying vulnerable groups, which can inform the development of targeted support and psychological services for students during future pandemics.

This study demonstrated that campus lockdown due to the COVID-19 pandemic had distinct effects on different emotional symptoms of university students. Specifically, we proved that average mental health progressively and significantly worsened from pre-pandemic to COVID-19 campus lockdowns, which was in line with two prior studies reporting impaired mental health in UK university students from before to during the COVID-19 pandemic (30, 31). Furthermore, previous longitudinal studies consistently reported elevated depression symptoms among university students during the pandemic compared to pre-pandemic (18, 20, 31–35), while our study further demonstrated that average depression symptom peaked at the initial COVID-19 outbreak (i.e., lockdown T1), and relieved after 1 month later (i.e., lockdown T2). Studies on anxiety symptoms of university students often reported mixed findings. Some studies reported increased anxiety symptoms during COVID-19 relative to pre-pandemic (18, 32–35), but others as well as this study showed that anxiety symptoms were stable before and during COVID-19 (20, 31, 36, 37). Regarding stress symptoms, evidence indicates that university students had higher stress levels during the pandemic compared to before (21, 30, 32, 38), while our results further showed that average stress symptoms improved from pre-pandemic to the initial COVID-19 outbreak and deteriorated 1 month later. Overall, our findings underlined the need to reinforce targeted prevention, surveillance, and accessible mental health care for university students at different stages of the pandemic lockdowns.

Although emerging evidence has suggested heterogeneous mental health responses to the pandemic (13, 14, 39), there is a lack of data on mental health trajectories among university students before and during COVID-19 campus lockdowns. Only one study, using piecewise latent growth models, reported stable mental health among 141 Dutch university students over a 3-month period, assessed before (Times 1–4) and during the COVID-19 lockdown (Times 5–8) with a 14 self-report item mental health continuum short-form (19). In contrast, our study found that the mental health of Chinese university students deteriorated during the COVID-19 campus lockdowns, identifying three distinct trajectories across three phases using LCMM. One possible explanation for the disparities in the mental health status of university students between our study and the previous one may be attributed to variations in assessment tools and data analysis methods employed. Additionally, differences in the COVID-19 epidemic situation, the implementation of campus lockdown measures, and pandemic control measures across various countries, cities, and universities could also contribute to these discrepancies. Furthermore, our study identified three distinct trajectories for depression and anxiety symptoms, as well as four distinct trajectories for stress symptoms. To our knowledge, this study provided the first evidence for the heterogeneity in the psychological impact of pandemic lockdowns on university students before and during the COVID-19 pandemic.

Further, students with fair or poor peer relationships were more likely to be in the vulnerable trajectories of depression, anxiety, and stress symptoms. Similarly, Sun et al. reported that perceived available peer support negatively contributed to depression symptoms among university students during the COVID-19 pandemic (40). Participants whose father had an education level of junior high school or below were found to be more vulnerable to experiencing deteriorating stress symptoms. Coincidentally, Villaume et al. found that adolescents from low parental education households had more negative changes in stress and mood in the COVID-19 pandemic (41).

Compared to previous studies employing either cross-sectional or longitudinal designs, this study contributes to the existing literature by unveiling the heterogeneity in the psychological impact of COVID-19 on university students before and during the pandemic lockdowns. It also sheds light on specific vulnerable subgroups that may necessitate tailored support during future crises. However, it is essential to acknowledge several limitations. First, our study employed a convenience sampling method and had a modest sample size, which could limit the generalizability of our findings to a broader population. Second, while GHQ-12 and DASS-21 have undergone validation in Chinese populations, it is essential to recognize China’s cultural and ethnic diversity. Our study focused on a specific group of participants, and the validation status may not fully encompass the richness of diversity across China. Third, the effectiveness of the LCMM utilized in this study might be compromised due to the limited availability of only three phases (42). Last, the relatively short duration between the second and third waves of data collection could limit the examination of the long-term effects of the lockdown on mental health.

In summary, this three-wave longitudinal study has provided valuable insights into the diverse landscape of mental health experiences among university students facing the COVID-19 pandemic campus lockdown. Our findings emphasize the significance of acknowledging and addressing this heterogeneity in mental health outcomes. It is imperative for universities to adopt a proactive approach in identifying and supporting vulnerable students while laying the groundwork for the development of more effective support systems and policies aimed at enhancing student well-being in preparation for future health crises. Additionally, there is a need for conducting long-term follow-up studies to assess the lasting impact of the post-pandemic period on the mental health of university students in future.

## Data availability statement

The raw data supporting the conclusions of this article will be made available by the authors, without undue reservation.

## Ethics statement

The studies involving humans were approved by the Institutional Review Board for Human Research Protections of Shanghai Jiao Tong University. The studies were conducted in accordance with the local legislation and institutional requirements. The participants provided their written informed consent to participate in this study.

## Author contributions

YQ: Conceptualization, Formal analysis, Funding acquisition, Methodology, Supervision, Visualization, Writing – original draft. ZL: Data curation, Formal analysis, Investigation, Project administration, Writing – review & editing. YZ: Data curation, Formal analysis, Investigation, Project administration, Writing – review & editing.
