# Supplementary material for: Changes in mental health among Chinese university students before and during campus lockdowns due to the COVID-19 pandemic: a three-wave longitudinal study
Source: Front Psychiatry. 2023 Nov 14;14:1267333. doi: 10.3389/fpsyt.2023.1267333 (PMC10682097; doi:10.3389/fpsyt.2023.1267333)
Supplement: Supplementary file 1 [file Table_1.docx]

**Supplementary Table 1** Goodness of fit indices of models with distinct latent classes.

| **Models with distinct number of latent classes** | **BIC** | **SABIC** | **Entropy** | **Proportion of each class (%)** | | | | | **Mean of posterior probabilities in each class (%)** | **Posterior probabilities >0.7 (%)** |
| --- | --- | --- | --- | --- | --- | --- | --- | --- | --- | --- |
|  |  |  |  | **1** | **2** | **3** | **4** | **5** |  |  |
| **Models for GHQ-12** | | | | | | | | |  |  |
| 1 | 3666 | 3644 | 1 | 100 | NA | NA | NA | NA | NA | NA |
| 2 | 3668 | 3630 | 0.793 | 14.63 | 85.37 | NA | NA | NA | 82.15, 95.7 | 66.67, 96.57 |
| **3** | **3665** | **3611** | **0.722** | **52.68** | **40.00** | **7.32** | **NA** | **NA** | **90.66, 84.91, 80.78** | **87.96, 86.59, 86.67** |
| 4 | 3667 | 3597 | 0.753 | 50.73 | 38.05 | 6.34 | 4.88 | NA | 87.12, 85.33, 84.02, 85.29 | 81.73, 87.18, 69.23, 70 |
| 5 | 3697 | 3612 | 0.748 | 14.63 | 9.76 | 20.00 | 43.90 | 11.71 | 80.66, 80.95, 68.79, 94.65, 80.80 | 76.67, 65, 46.34, 94.44, 70.83 |
| **Models for DASS-21 Depression** | | | | | | | | |  |  |
| 1 | 4034 | 4012 | 1 | 100 | NA | NA | NA | NA | NA | NA |
| 2 | 4015 | 3977 | 0.893 | 6.34 | 93.66 | NA | NA | NA | 85.07, 98.02 | 84.62, 97.40 |
| **3** | **4009** | **3955** | **0.652** | **25.37** | **38.05** | **36.59** | **NA** | **NA** | **85.07, 87.65, 81.74** | **80.77, 89.74, 72** |
| 4 | 4048 | 3978 | 0.790 | 46.34 | 46.34 | 2.93 | 4.39 | NA | 87.41, 88.27, 94.06, 93.96 | 86.32, 85.26, 100, 100 |
| 5 | 4078 | 3992 | 0.672 | 11.22 | 44.39 | 36.10 | 0 | 8.29 | 65.51, 87.42, 69.49, NA, 92.03 | 30.43, 87.91, 55.41, NA, 88.24 |
| **Models for DASS-21 Anxiety** | | | | | | | | |  |  |
| 1 | 3688 | 3666 | 1 | 100 | NA | NA | NA | NA | NA | NA |
| 2 | 3635 | 3597 | 0.645 | 6.34 | 93.66 | NA | NA | NA | 93.83, 88.64 | 91.89, 90.08 |
| **3** | **3603** | **3549** | **0.790** | **25.37** | **38.05** | **36.59** | **NA** | **NA** | **92.37, 90.65, 94.56** | **87.69, 90.77, 100** |
| 4 | 3614 | 3544 | 0.708 | 46.34 | 46.34 | 2.93 | 4.39 | NA | 76.47,82.48, 88.79, 96.18 | 66.04, 76.71, 81.16, 100 |
| 5 | 3618 | 3532 | 0.708 | 11.22 | 44.39 | 36.10 | 0.00 | 8.29 | 90.94, 75.82, 81.73, 80.60, 85.92 | 91.67, 60.53, 71.43, 71.43, 84.62 |
| **Models for DASS-21 Stress** | | | | | | | | |  |  |
| 1 | 3987 | 3965 | 1 | 100 | NA | NA | NA | NA | NA | NA |
| 2 | 3986 | 3948 | 0.462 | 31.22 | 68.78 | NA | NA | NA | 85.23, 83.56 | 78.12, 88.65 |
| 3 | 3996 | 3942 | 0.698 | 47.80 | 47.80 | 4.39 | NA | NA | 86.65, 83.34, 92.54 | 84.69, 75.51, 100 |
| **4** | **3976** | **3906** | **0.684** | **48.29** | **22.44** | **16.10** | **13.17** | **NA** | **80.09, 81.93, 84.42, 88.58** | **71.72, 80.43, 84.85, 85.19** |
| 5 | 4010 | 3924 | 0.756 | 3.41 | 43.41 | 13.66 | 33.17 | 6.34 | 79.69, 82.32, 72.30, 91.16, 91.95 | 71.43, 80.90, 57.14, 89.71, 92.31 |

Bold: The final model we selected.

BIC: Bayesian information criteria; SABIC: Sample size-adjusted BIC.

Grid search applying 50 iterations from 100 random vectors of initial values.

**Supplementary Table 2** Associations between sociodemographic and distinct trajectories of GHQ-12.

| **Variables** | **Consistently fair** | **Deteriorating at lockdown T2** |
| --- | --- | --- |
|  | **aOR (95% CI)** | **aOR (95% CI)** |
| Gender |  |  |
| Male | - | - |
| Female | 0.90 (0.44-1.81) | 1.09 (0.30-3.93) |
| BMI |  |  |
| Normal weight | - | - |
| Abnormal weight^a^ | 0.62 (0.27-1.41) | 1.05 (0.27-4.16) |
| Grade |  |  |
| Undergraduate | - | - |
| Postgraduate | 0.69 (0.25-1.94) | 0.66 (0.07-6.76) |
| Major |  |  |
| Engineering | - | - |
| Else^b^ | 0.60 (0.29-1.23) | 2.38 (0.64-8.82) |
| Peer relationships |  |  |
| Good | - | - |
| Fair or poor | 5.79 (2.87-11.69)*** | 1.93 (0.54-6.82) |
| Family residence |  |  |
| North | - | - |
| South | 1.05 (0.50-2.19) | 1.88 (0.49-7.24) |
| Only-child family |  |  |
| No | - | - |
| Yes | 0.84 (0.39-1.78) | 0.81 (0.22-2.98) |
| Father’s education level |  |  |
| College or above | - | - |
| High School | 1.77 (0.67-4.68) | 0.45 (0.06-3.54) |
| Junior high school or below | 4.90 (1.21-19.83)* | 0.46 (0.02-11.30) |
| Mother’s education level |  |  |
| College or above | - | - |
| High School | 0.53 (0.21-1.31) | 2.26 (0.43-11.91) |
| Junior high school or below | 0.29 (0.07-1.17) | 0.58 (0.02-14.11) |
| Annual household income (CNY) |  |  |
| 80,000 - 300,000 | - | - |
| < 80,000 | 1.18 (0.44-3.16) | 3.14 (0.38-26.29) |
| > 300,000 | 0.33 (0.13-0.87)* | 4.08 (1.03-16.22)* |

Notes: The “consistently good” class is set as the reference group.

– indicates the reference group.

^a^ Abnormal weight includes underweight, overweight, and obesity.

^b^ Else includes liberal arts, science, and medicine.

* *p* < 0.05; *** *p* < 0.001.

**Supplementary Table 3** Associations between sociodemographic and distinct trajectories of DASS-21 depression symptoms.

| **Variables** | **Deteriorating** | **Recovery** |
| --- | --- | --- |
|  | **aOR (95% CI)** | **aOR (95% CI)** |
| Gender |  |  |
| Male | - | - |
| Female | 1.13 (0.48-2.63) | 0.84 (0.40-1.74) |
| BMI |  |  |
| Normal weight | - | - |
| Abnormal weight^a^ | 0.98 (0.37-2.57) | 0.82 (0.35-1.90) |
| Grade |  |  |
| Undergraduate | - | - |
| Postgraduate | 0.73 (0.20-2.60) | 0.63 (0.21-1.83) |
| Major |  |  |
| Engineering | - | - |
| Else^b^ | 1.41 (0.61-3.28) | 1.08 (0.52-2.23) |
| Peer relationships |  |  |
| Good | - | - |
| Fair or poor | 12.22 (5.01-29.80)*** | 5.19 (2.41-11.18)*** |
| Family residence |  |  |
| North | - | - |
| South | 2.12 (0.86-5.21) | 0.82 (0.39-1.72) |
| Only-child family |  |  |
| No | - | - |
| Yes | 1.43 (0.55-3.67) | 0.75 (0.35-1.64) |
| Father’s education level |  |  |
| College or above | - | - |
| High School | 1.18 (0.34-4.10) | 1.51 (0.56-4.10) |
| Junior high school or below | 2.02 (0.41-10.01) | 1.68 (0.42-6.70) |
| Mother’s education level |  |  |
| College or above | - | - |
| High School | 0.50 (0.16-1.54) | 0.80 (0.32-2.01) |
| Junior high school or below | 0.50 (0.09-2.63) | 0.66 (0.17-2.61) |
| Annual household income (CNY) |  |  |
| 80,000 - 300,000 | - | - |
| < 80,000 | 1.28 (0.39-4.23) | 0.70 (0.25-1.98) |
| > 300,000 | 0.51 (0.16-1.60) | 0.89 (0.36-2.23) |

Notes: The “consistently good” class is set as the reference group.

– indicates the reference group.

^a^ Abnormal weight includes underweight, overweight, and obesity.

^b^ Else includes liberal arts, science, and medicine.

*** *p* < 0.001.

**Supplementary Table 4** Associations between sociodemographic and distinct trajectories of DASS-21 anxiety symptoms.

| **Variables** | **Consistently poor** | **Poor and deteriorating at lockdown T1** |
| --- | --- | --- |
|  | **aOR (95% CI)** | **aOR (95% CI)** |
| Gender |  |  |
| Male | - | - |
| Female | 0.90 (0.46-1.75) | 0.21 (0.04-1.21) |
| BMI |  |  |
| Normal weight | - | - |
| Abnormal weight^a^ | 1.57 (0.72-3.39) | 3.53 (0.75-16.70) |
| Grade |  |  |
| Undergraduate | - | - |
| Postgraduate | 0.92 (0.32-2.63) | 0.46 (0.04-5.31) |
| Major |  |  |
| Engineering | - | - |
| Else^b^ | 0.93 (0.48-1.81) | 0.80 (0.17-3.70) |
| Peer relationships |  |  |
| Good | - | - |
| Fair or poor | 3.49 (1.79-6.78)*** | 4.85 (1.02-23.20)* |
| Family residence |  |  |
| North | - | - |
| South | 1.37 (0.68-2.73) | 0.66 (0.14-3.16) |
| Only-child family |  |  |
| No | - | - |
| Yes | 0.96 (0.46-2.00) | 0.56 (0.12-2.53) |
| Father’s education level |  |  |
| College or above | - | - |
| High School | 1.42 (0.54-3.73) | 0.46 (0.04-5.32) |
| Junior high school or below | 2.31 (0.65-8.20) | 3.02 (0.30-30.37) |
| Mother’s education level |  |  |
| College or above | - | - |
| High School | 0.74 (0.30-1.81) | 3.30 (0.50-21.82) |
| Junior high school or below | 0.71 (0.19-2.63) | 0.52 (0.03-8.67) |
| Annual household income (CNY) |  |  |
| 80,000 - 300,000 | - | - |
| < 80,000 | 1.11 (0.43-2.88) | 1.29 (0.20-8.44) |
| > 300,000 | 1.19 (0.50-2.83) | 2.47 (0.37-16.72) |

Notes: The “consistently good” class is set as the reference group.

– indicates the reference group.

^a^ Abnormal weight includes underweight, overweight, and obesity.

^b^ Else includes liberal arts, science, and medicine.

* *p* < 0.05; *** *p* < 0.001.

**Supplementary Table 5** Associations between sociodemographic and distinct trajectories of DASS-21 stress symptoms.

| **Variables** | **Consistently good** | **Poor but alleviation at lockdown T1** | **Deteriorating** |
| --- | --- | --- | --- |
|  | **aOR (95% CI)** | **aOR (95% CI)** | **aOR (95% CI)** |
| Gender |  |  |  |
| Male | - | - | - |
| Female | 1.24 (0.56-2.73) | 2.51 (0.86-7.30) | 1.52 (0.51-4.56) |
| BMI |  |  |  |
| Normal weight | - | - | - |
| Abnormal weight^a^ | 0.76 (0.32-1.77) | 0.31 (0.08-1.18) | 0.33 (0.08-1.33) |
| Grade |  |  |  |
| Undergraduate | - | - | - |
| Postgraduate | 0.90 (0.31-2.67) | 0.14 (0.01-1.46) | 0.54 (0.11-2.56) |
| Major |  |  |  |
| Engineering | - | - | - |
| Else^b^ | 0.95 (0.43-2.09) | 1.23 (0.43-3.51) | 0.85 (0.28-2.55) |
| Peer relationships |  |  |  |
| Good | - | - | - |
| Fair or poor | 2.90 (1.22-6.88)* | 6.75 (2.28-20.00)*** | 6.10 (1.92-19.37)** |
| Family residence |  |  |  |
| North | - | - | - |
| South | 0.91 (0.41-2.03) | 1.08 (0.37-3.13) | 1.78 (0.38-3.62) |
| Only-child family |  |  |  |
| No | - | - | - |
| Yes | 0.71 (0.30-1.66) | 1.23 (0.39-3.86) | 1.76 (0.50-6.25) |
| Father’s education level |  |  |  |
| College or above | - | - | - |
| High School | 1.34 (0.45-3.99) | 2.78 (0.66-11.83) | 1.27 (0.26-6.31) |
| Junior high school or below | 2.39 (0.50-11.35) | 5.24 (0.62-44.22) | 10.00 (1.39-71.64)* |
| Mother’s education level |  |  |  |
| College or above | - | - | - |
| High School | 0.75 (0.28-1.99) | 0.73 (0.19-2.81) | 1.03 (0.26-4.15) |
| Junior high school or below | 0.37 (0.08-1.61) | 0.34 (0.04-2.78) | 0.32 (0.04-2.50) |
| Annual household income (CNY) |  |  |  |
| 80,000 - 300,000 | - | - | - |
| < 80,000 | 1.25 (0.41-3.79) | 0.80 (0.17-3.81) | 1.18 (0.27-5.12) |
| > 300,000 | 1.94 (0.70-5.38) | 1.32 (0.34-5.12) | 0.31 (0.03-2.93) |

Notes: The “consistently very good” class is set as the reference group.

– indicates the reference group.

^a^ Abnormal weight includes underweight, overweight, and obesity.

^b^ Else includes liberal arts, science, and medicine.

* *p* < 0.05; ** *p* < 0.01; *** *p* < 0.001.
